# Ratio of Two Independent Lindley Random Variables

**DOI:** 10.1007/s44199-022-00050-4

**Published:** 2022-10-15

**Authors:** Mohammad Shakil, Aneeqa Khadim, Aamir Saghir, Mohammad Ahsanullah, B. M. Golam Kibria, M. Ishaq Bhatti

**Affiliations:** 1 Department of Mathematics, Miami Dade College, Hialeah, FL USA; 2 Department of Mathematics, Mirpur University of Science and Technology, Mirpur, AJK Pakistan; 3 Department of Mathematics, Mirpur University of Science and Technology, Mirpur, AJK 10250 Pakistan; 4 Department of Management Sciences, Rider University, Lawrenceville, NJ USA; 5 Department of Mathematics and Statistics, Florida International University, Miami, USA; 6 La Trobe Business School, La Trobe University, La Trobe, Australia

**Keywords:** Lindley distribution, Characterizations, Estimation, Ratio of independent random variables, 33B99, 33C90, 33D90, 33E99, 62E15

## Abstract

The distribution of the ratio of two independently distributed Lindley random variables $X$ and $Y$, with different parameters, is derived. The associated distributional properties are provided. Furthermore, the proposed ratio distribution is fitted to two applications data (COVID-19 and Bladder Cancer Data), and compared it with some well-known right-skewed variations of Lindley distribution, namely; Lindley distribution, new generalized Lindley distribution, new quasi Lindley distribution and a three parameter Lindley distribution. The numerical result of the study reveals that the proposed distribution of two independent Lindley random variables fits better to the above said data sets than the compared distribution.

## Introduction and Motivation

For modeling lifetime data and studying stress-strength problems, Lindley [17] introduced a positively-skewed distribution for a non-negative continuous random variable. It is defined as a mixture of exponential and gamma distributions, and is well known in the literature as Lindley distribution. In recent years, there has been a great interest by many authors and researchers in the study of the Lindley distribution and its applications to model failure time data with increasing, decreasing, unimodal and bathtub shaped hazard rates. For details on Lindley distribution and its extension, the interested readers are referred to Lindley [17], Ghitany et al. [12], Mazucheli and Achcar [21], Al-Mutairi et al. [6], Cakmakyapan and Kadilar [7], Tomy [38], and references therein.

Several lifetime distributions have been proposed in statistics literature to model the survival data. Lindley distribution is one of them [4]. The distributions of the ratio $Z\; = \;\left| \frac{X}{Y} \right|$ of two independently distributed random variables $X$ and $Y$, when they belong to the same family, is of great interest in many problems of applied sciences. As pointed out by Nadarajah and Gupta [24], “the distribution of the ratio is of interest in biological and physical sciences, econometrics, and ranking and selection. Examples include Mendelian inheritance ratios in genetics, mass to energy ratios in nuclear physics, target to control precipitation in meteorology, and inventory ratios in economics”. It has been extensively studied by many authors and researchers, among them, Marsaglia [20], Lee et al. [18], Korhonen and Narula [16], Press [28], Pham-Gia [27], Nadarajah [22], Nadarajah and Gupta [24], Nadarajah and Kotz. [25], Ali et al. [5], are notable.

It appears from literature that no attention has been paid in details to the distribution of the ratio of two independent Lindley random variables. As stated above, motivated by the importance of the distributions of the ratio of two independent random variables in many applied fields, in this paper, we derive the exact distribution of the ratio $Z\; = \;\left| \frac{X}{Y} \right|$ of two independently distributed Lindley random variables $X$ and $Y$ with different parameters $\alpha$ and $\beta$, respectively. The newly proposed distribution of the ratio $Z\; = \;\left| \frac{X}{Y} \right|$ has two different parameters $\alpha$ and $\beta$, as stated above. It discusses several statistical properties, along with estimation of parameters and applications to two real lifetime datasets, namely, COVID-19 and bladder cancer, to illustrate the importance of the proposed distribution, which is compared with some known variations of Lindley distributions, namely, the LD Lindley [17], the NGLD Elbatal et al. [9], the NQLD Shanker and Ghebretsadik [35] and ATPLD Shanker et al. [36].

The organization of this paper is divided into different sections as follows. Section 2 contains the proposed distribution of the ratio $Z\; = \;\left| \frac{X}{Y} \right|$ of two independently distributed Lindley random variables, along with several distributional properties. Since characterizations play important roles in distribution theory, some characterizations of the proposed distribution based on truncated moments are given in Sect. 3. The estimation of parameters and applications to two real datasets are provided in Sects. 4 and 5, respectively. Finally, some concluding remarks are given in Sect. 6. Since the derivations of the proposed ratio distribution involve several special functions and formulas, these are provided in Appendix I.

## Distribution of the Ratio $Z\; = \;\left| \frac{X}{Y} \right|$

Let $$X$$ and $$Y$$ be two independently distributed Lindley random variables with different parameters $$\alpha$$ and $$\beta$$. Then their $$pdfs$$$$f_{X} (x)$$ and $$f_{Y} (y)$$ are given as follows:

1 $$f_{X} (x)\; = \;\left( {\frac{{\alpha^{2} }}{\alpha \; + \;1}} \right)\,\left( {1\; + \;x} \right)\,e^{ - \,\alpha \,x} \,,\;x > 0,\,\,\alpha > 0,$$

and

2 $$f_{Y} (y)\; = \;\left( {\frac{{\beta^{2} }}{\beta \; + \;1}} \right)\,\left( {1\; + \;y} \right)\,e^{ - \,\beta \,y} \,,\;y > 0,\,\,\beta > 0.$$

The corresponding $cdfs$ are given as follows:

3 $$F_{X} \left( x \right)\; = \;P\,\left( {X \le x} \right)\; = \;1\; - \;\left( {\frac{\alpha \; + \;1\; + \;\alpha \,x}{{\alpha \; + \;1}}} \right)\,\,e^{ - \,\alpha \,x} \,,\;x > 0,\,\,\alpha > 0,$$

and

4 $$F_{Y} (y) = \;P\,\left( {Y \le y} \right)\; = \;1\; - \;\left( {\frac{\beta \; + \;1\; + \;\beta \,y}{{\beta \; + \;1}}} \right)\,\,e^{ - \,\beta \,y} \,,\;y > 0,\,\,\beta > 0.$$

### CDF of the Ratio $Z\; = \;\left| \frac{X}{Y} \right|$

In what follows, we derive the explicit expressions for the $cdf$ of $Z\; = \;\left| \frac{X}{Y} \right|$ in Theorem 1.

#### Theorem 1

Suppose $X$ is a Lindley random variable with $pdf$ (1) and $cdf$ (3). Also, suppose $Y$ is another Lindley random variable with $pdf$ (2) and $cdf$ (4). Then the $cdf$ of the ratio $Z = \left| \frac{X}{Y} \right|$ is given by

5 $$F_{Z} \,\left( z \right)\; = \;1\; - \;\left( {\frac{{\beta^{2} }}{\beta \; + \;1}} \right)\,\frac{1}{{\left( {\alpha \,z\; + \;\beta } \right)}}\left[ {1\; + \;\frac{1}{{\left( {\alpha \,z\; + \;\beta } \right)}} + \frac{\alpha \,z}{{\left( {\alpha \; + \;1} \right)\,\left( {\alpha \,z\; + \;\beta } \right)}} + \;\frac{2\,\alpha \,z}{{\left( {\alpha \; + \;1} \right)\,\left( {\alpha \,z\; + \;\beta } \right)^{2} }}} \right]\;.$$

#### Proof

Using (3) for the $cdf$ of Lindley random variable $X$ and (2) for the $pdf$ of Lindley random variable $Y$, the $cdf$ of the ratio $Z = \left| \frac{X}{Y} \right|$ is given as

6 $$\begin{aligned} F_{Z} \left( z \right) & = \Pr \left( {\left| \frac{X}{Y} \right| \le z} \right) \\ & = \Pr \left( {\left| X \right| \le z\,\left| Y \right|} \right) = \int\limits_{0}^{\infty } {F_{X} \left( {z\,y} \right)\;f_{Y} (y)\;dy} \\ & = \;\int\limits_{0}^{\infty } {\left[ {1\; - \;\left( {\frac{\alpha \; + \;1\; + \;\alpha \,z\,y}{{\alpha \; + \;1}}} \right)\,\,e^{ - \,\alpha \,z\,y} } \right]\,\left\{ {\left( {\frac{{\beta^{2} }}{\beta \; + \;1}} \right)\,\,\left( {1\; + \;y} \right)\,e^{ - \,\beta \,y} } \right\}} \,dy\,\quad \;\;\quad \;\;\;\quad \quad \quad \quad \quad \quad \quad \quad \quad \quad \quad \quad \quad \quad \quad \quad \quad \quad \; \\ & = \;\int\limits_{0}^{\infty } {\left[ {\left( {\frac{{\beta^{2} }}{\beta \; + \;1}} \right)\,\,\left( {1\; + \;y} \right)\,e^{ - \,\beta \,y} \; - \;\left( {\frac{{\beta^{2} }}{\beta \; + \;1}} \right)\,\left( {\frac{\alpha \; + \;1\; + \;\alpha \,z\,y}{{\alpha \; + \;1}}} \right)\,\,\left( {1\; + \;y} \right)\,e^{{ - \,\left( {\alpha \,z\; + \;\beta } \right)\,y}} } \right]\,} \,dy\,. \\ \end{aligned}$$

In the right-hand side of the last Eq. (6), we note that the first integral is equal to 1 as integrand is the $pdf$ (2) of Lindley random variable $Y$. Thus, by substituting $\left( {\alpha \,z\; + \;\beta } \right)\,y\; = \;t$ in (6), integrating it with respect to $t$ from $t\; = \;0$ to $t\; = \;\infty$, using Lemma A.1, and then simplifying, the proof of Theorem 1 easily follows.

### pdf of the Ratio $Z = \left| \frac{X}{Y} \right|$

In this subsection, we derive the explicit expressions for the $pdf$ of $Z\; = \;\left| \frac{X}{Y} \right|$ in Theorem 2.

#### Theorem 2

Suppose $X$ is a Lindley random variable with $pdf$ (1) and $cdf$ (3). Also, suppose $Y$ is another Lindley random variable with $pdf$ (2) and $cdf$ (4). Then the $pdf$ of the ratio $Z = \left| \frac{X}{Y} \right|$ is given by

7 $$f_{Z} \,\left( z \right)\; = \;\left( {\frac{{\alpha^{2} }}{\alpha \; + \;1}} \right)\,\,\left( {\frac{{\beta^{2} }}{\beta \; + \;1}} \right)\,\,\left[ {\frac{1}{{\left( {\alpha \,z\; + \;\beta } \right)^{2} }}\; + \;\frac{{2\,\left( {z\; + \;1} \right)}}{{\,\left( {\alpha \,z\; + \;\beta } \right)^{3} }}\; + \;\frac{6\,z}{{\,\left( {\alpha \,z\; + \;\beta } \right)^{4} }}} \right].$$

#### Proof

The pdf of $Z = \left| \frac{X}{Y} \right|$ can be expressed as

8 $$\begin{aligned} f_{Z} \,\left( z \right) & = \int\limits_{0}^{\infty } {y\,f_{X} \left( {z\,y\,} \right)\;f_{Y} (y)\;dy} \\ & = \,\;\left( {\frac{{\alpha^{2} }}{\alpha \; + \;1}} \right)\,\,\left( {\frac{{\beta^{2} }}{\beta \; + \;1}} \right)\;\int\limits_{0}^{\infty } {y\,\left( {1\; + \;z\,y} \right)\,\left( {1\; + \;y} \right)\,e^{{ - \,\left( {\alpha \,z\; + \;\beta } \right)\,y}} } \,dy \\ & = \,\;\left( {\frac{{\alpha^{2} }}{\alpha \; + \;1}} \right)\,\,\left( {\frac{{\beta^{2} }}{\beta \; + \;1}} \right)\;\int\limits_{0}^{\infty } {\left[ {y\; + \;\left( {z\; + \;1} \right)\,y^{2} \; + \;z\,y^{3} } \right]\,e^{{ - \,\left( {\alpha \,z\; + \;\beta } \right)\,y}} } \,dy. \\ \end{aligned}$$

By substituting $\left( {\alpha \,z\; + \;\beta } \right)\,y\; = \;t$ in (8), integrating it with respect to $t$ from $t\; = \;0$ to $t\; = \;\infty$, using Lemma A.1, and then simplifying, the proof of Theorem 2 easily follows.

### Plots of the pdf and cdf of the Ratio $Z = \left| \frac{X}{Y} \right|$

**Fig. 1:**
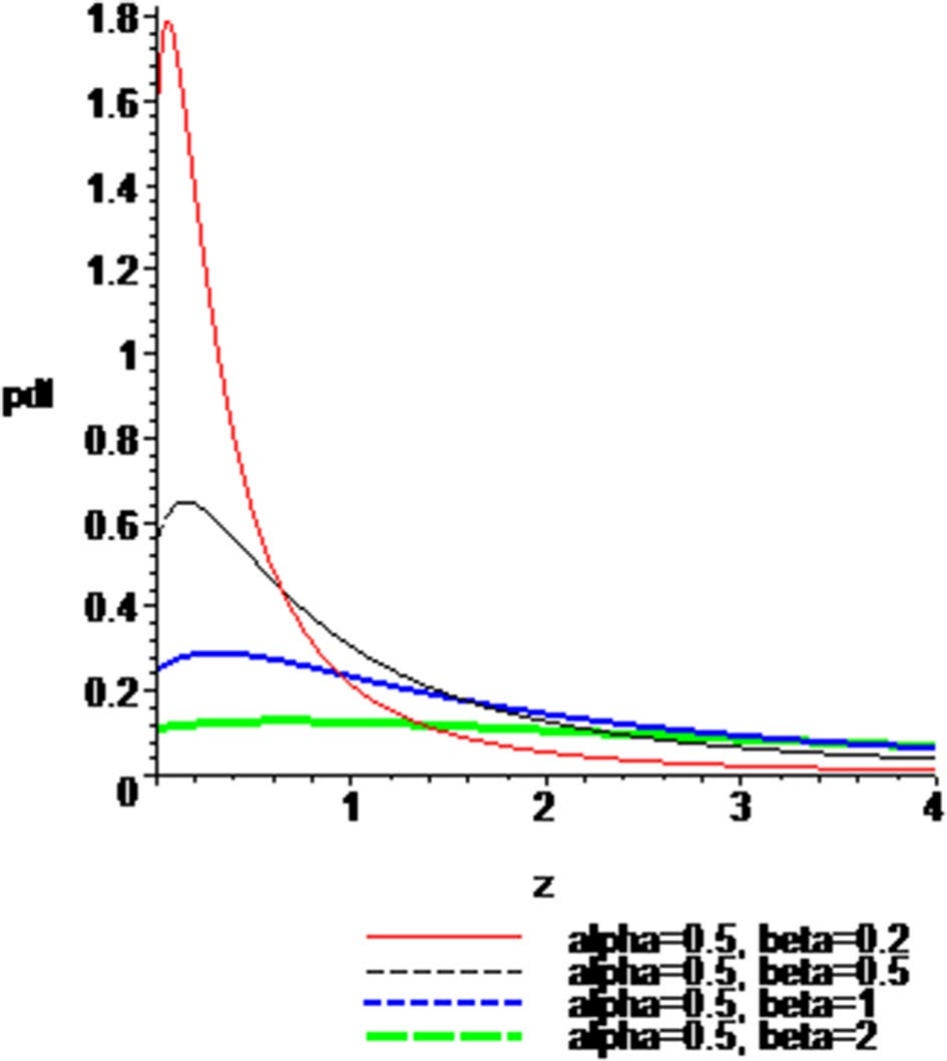
$$pdf$$, when $$\alpha \; = \;0.5$$ and $$\beta \; = \;0.2,\;0.5,\;1,\;2$$

**Fig. 2:**
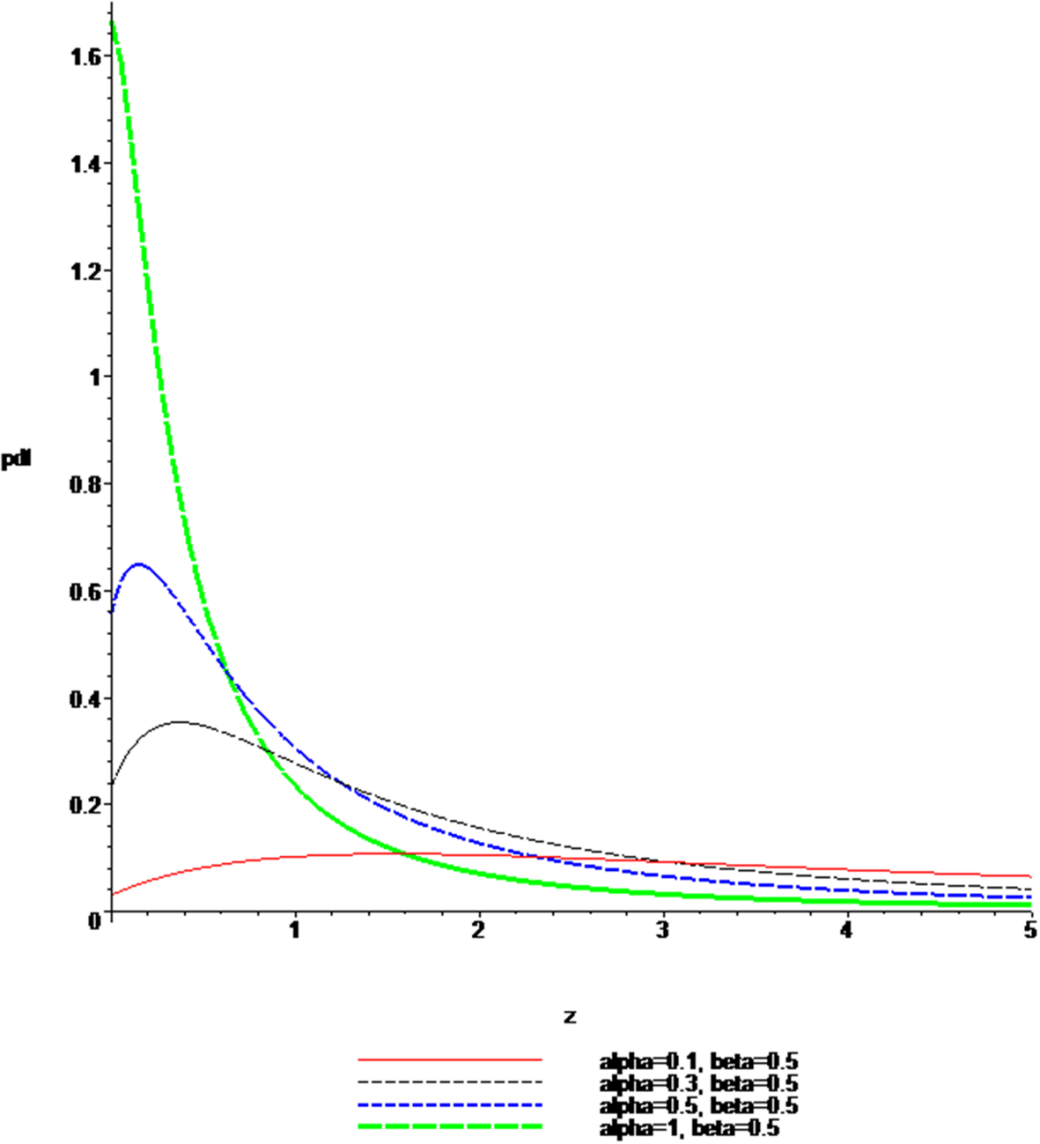
$$pdf$$, when $$\alpha \; = \;0.1,\;0.3,\;0.5,\;1$$ and $$\beta \; = \;0.5$$

**Fig. 3:**
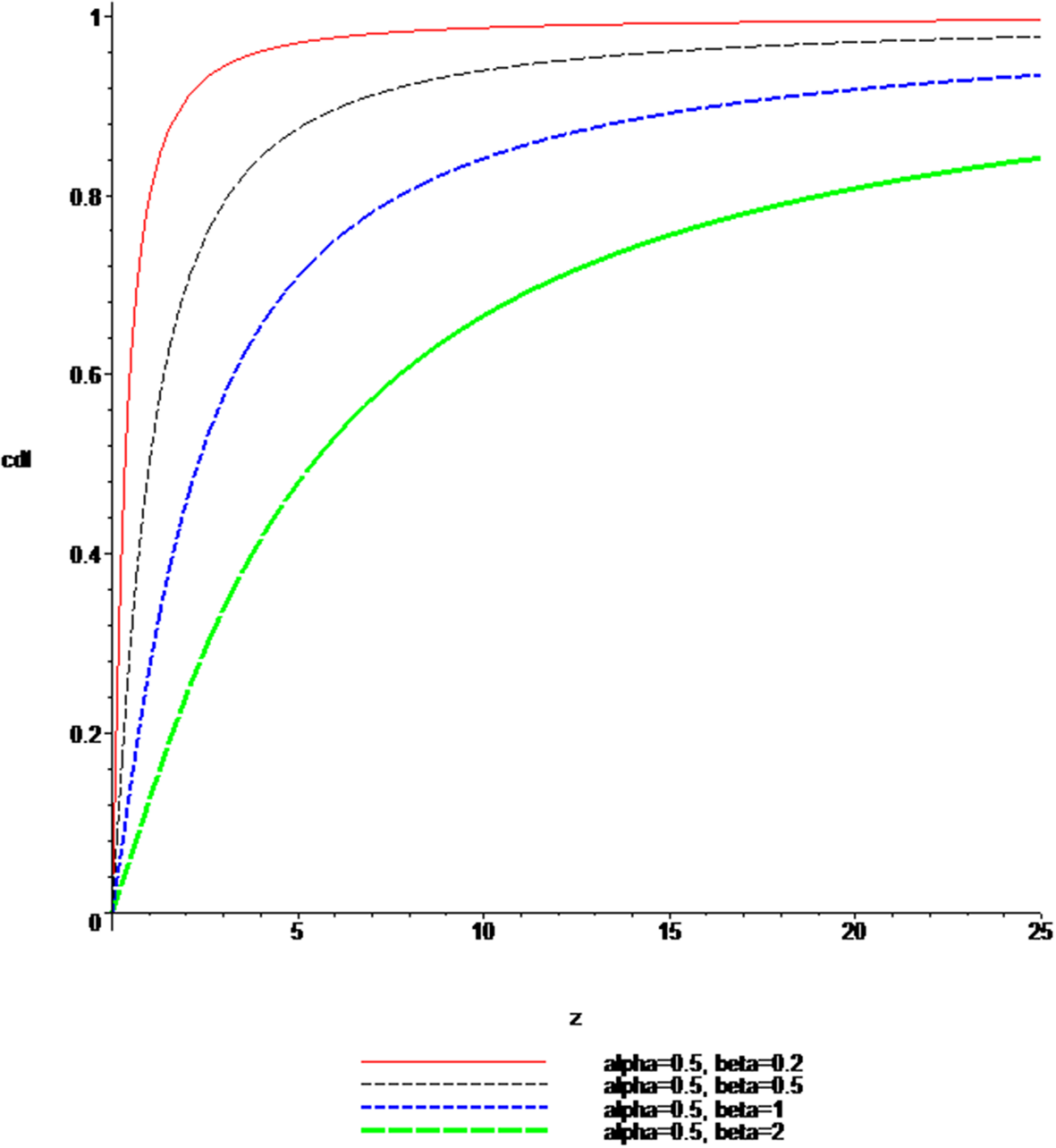
$$cdf$$, when $$\alpha \; = \;0.5$$ and $$\beta \; = \;0.2,\;0.5,\;1,\;2$$

**Fig. 4:**
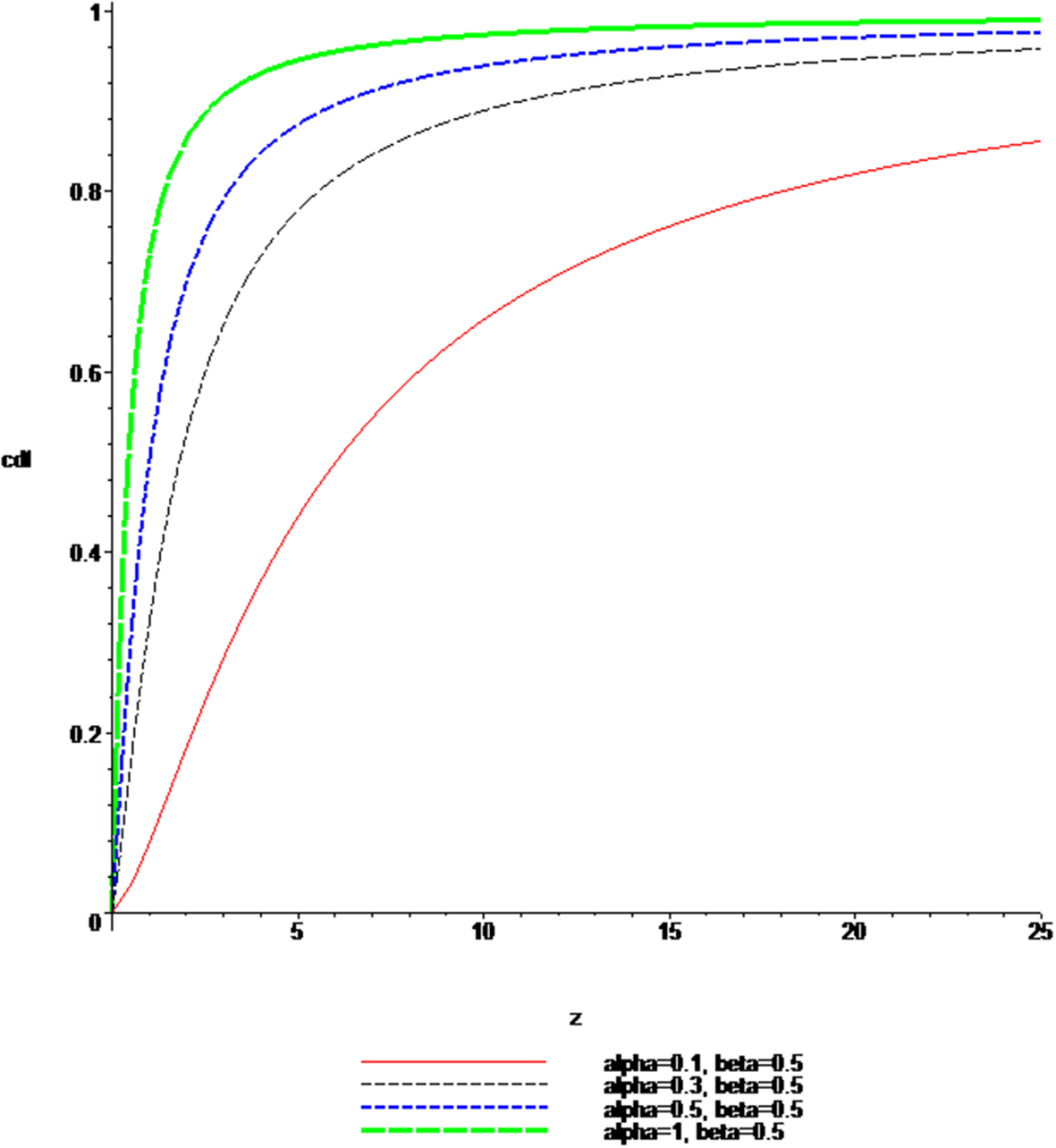
$$cdf$$, when $$\alpha \; = \;0.1,\;0.3,\;0.5,\;1$$ and $$\beta \; = \;0.5$$

The possible shapes of the $pdf$ (7) and $cdf$ (5) of the ratio for $\alpha \; = \;0.5$ and different values of $\beta$, and for $\beta = 0.5$ and different values of $\alpha$, are provided in Figs. 1, 2, 3 and 4, respectively. The effects of the parameters are evident from these graphs. In view of these graphs, the proposed distribution appears to be unimodal and right skewed.

### Moments

In this subsection, the expressions for the moments of RV $Z = \left| \frac{X}{Y} \right|$ have been derived. We derive the $kth$ moment of RV $Z = \left| \frac{X}{Y} \right|$ in terms of beta function, where $- 1\; < \;k\; < \;1$. It will be noted that only the fractional moments of order $- 1\; < \;k\; < \;1$ of Z exist.

#### Theorem 3

If $Z$ is a random variable with pdf given by (7), then its $kth$ moment can be expressed as

9 $$\begin{gathered} E\,\left( {Z^{k} } \right) = \left( {\frac{1}{\alpha \; + \;1}} \right)\,\,\left( {\frac{1}{\beta \; + \;1}} \right)\,\,\left( {\frac{\beta }{\alpha }} \right)^{k} \,\left[ {\,\alpha \,\left( {\beta \; + \;2} \right)B\,\left( {k\; + \;1\,,\;1 - k} \right)} \right. \hfill \\ \quad \quad \quad \quad \quad \quad \quad \quad \quad \quad \quad \quad \quad \quad \left. { + \;2\,\beta \,B\,\left( {k\; + \;2\,,\;1 - k} \right)\; + \;6\,B\,\left( {k\; + \;2\,,\;2 - k} \right)\,} \right], \hfill \\ \end{gathered}$$

where $- 1\; < \;k\; < \;1$ and $B\,\left( . \right)$, denotes Beta function (or Euler’s function of the first kind).

#### Proof

We have

10 $$E\,\left( {Z^{k} } \right) = \;\int\limits_{0}^{\infty } {z^{k} } \left( {\frac{{\alpha^{2} }}{\alpha \; + \;1}} \right)\,\,\left( {\frac{{\beta^{2} }}{\beta \; + \;1}} \right)\,\,\left[ {\frac{1}{{\left( {\alpha \,z\; + \;\beta } \right)^{2} }}\; + \;\frac{{2\,\left( {z\; + \;1} \right)}}{{\,\left( {\alpha \,z\; + \;\beta } \right)^{3} }}\; + \;\frac{6\,z}{{\,\left( {\alpha \,z\; + \;\beta } \right)^{4} }}} \right]\;dz.$$

**Fig. 5:**
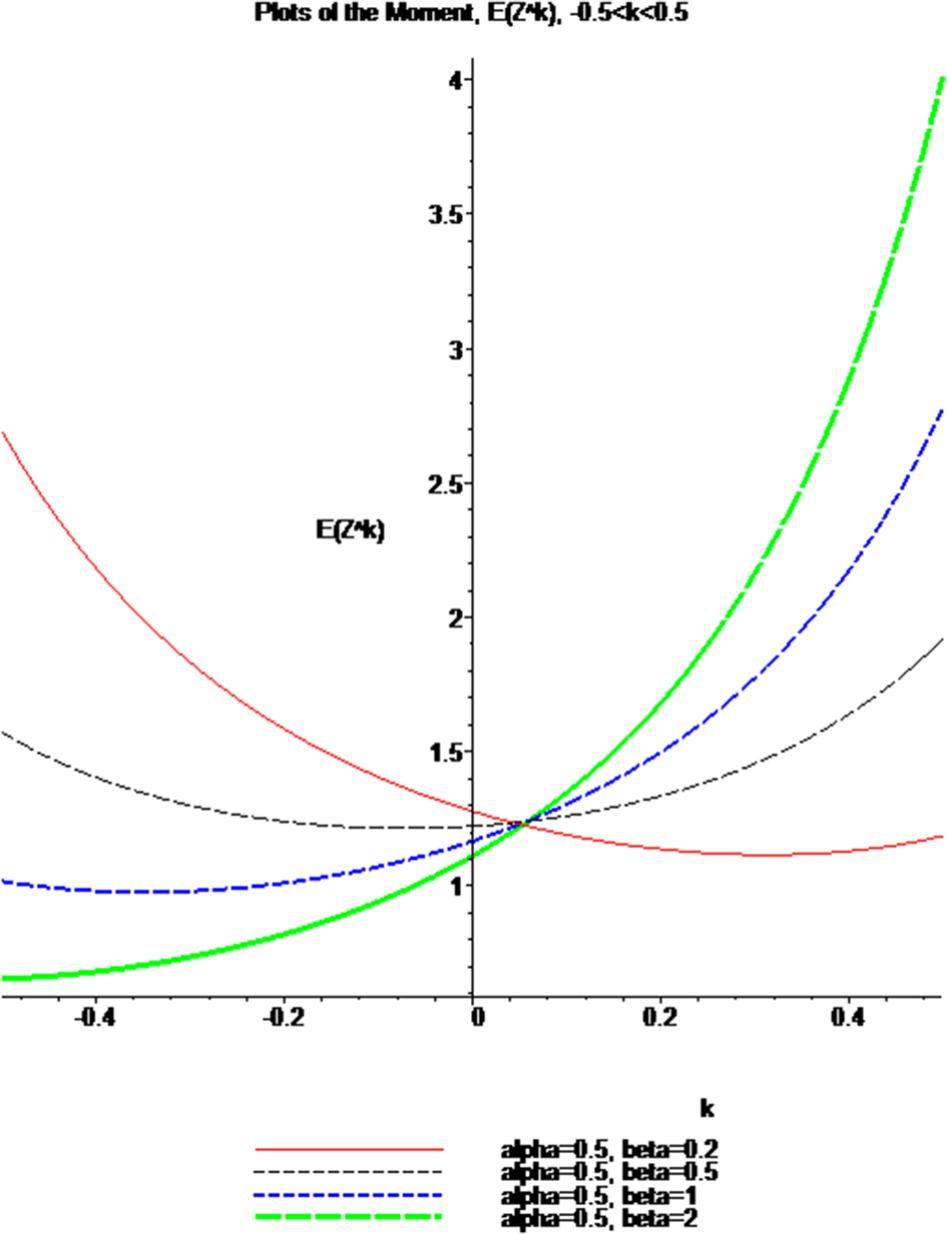
Plots of the moment, $E\;\left( {Z^{k} } \right)$, when $- 0.5\; \le \;k\; \le \;0.5$

Using Lemma A.2 (Appendix II) in (10), integrating and simplifying, the Theorem 3 easily follows, provided $- 1\; < \;k\; < \;1$. It is evident from Theorem 3 that only the fractional moments of order $- 1\; < \;k\; < \;1$ of Z exist. For a recent nice paper on fractional moments of any real order $p$ of any real-valued random variable $X$ with $E\left( {\left| X \right|^{p} } \right)\; < \;\infty$, and, in particular, for the fractional moments of a Poisson distribution (see, Pinelis [29]. The interested readers are also referred to Ahsanullah et al. [2], Shakil and Kibria [31], and Shakil et al. [32], where the authors have developed some continuous probability distributions with fractional moments. As pointed out by Ahsanullah et al. [2], one of the earliest examples on calculating the non-integer moments (NIM) was related to the spans of random walks. More recently, the properties of non-integer moments have found application in the study of random resistor networks, chaos, and diffusion-limited aggregation (see Weiss et al. (39), and references therein). Also, one is referred to Consortini and Rigal [8] and Innocenti and Consortini [14] for the usefulness of fractional moments in atmospheric laser scintillation. Using the software Maple, and Eq. (8), the graphs of the moment, $E\;\left( {Z^{k} } \right)$, when $- 0.5\; \le \;k\; \le \;0.5$, for some values of the parameter, are sketched in Fig. 5.

From Fig. 5, it is observed that, for $- 0.5\; \le \;k\; \le \;0.5$, the moment, $E\;\left( {Z^{k} } \right)$, is a concave up function of $k$, for the given values of parameters, and, obviously, as $k\; \to \; \pm 1$, $E\;\left( {X^{k} } \right)\; \to \;\infty$.

### Entropy of the RV $Z = \left| \frac{X}{Y} \right|$

The entropy of a continuous random variable $Z$ is a measure of variation of uncertainty and has applications in many fields such as physics, engineering and economics, among others. According to Shannon [37], the entropy measure of a continuous real random variable $Z$ is given by

11 $$E\,\left( { - \ln f_{Z} (z)} \right) = - \int\limits_{0}^{\infty } {\left[ {\ln f_{Z} (z)} \right]\;f_{Z} (z)\;dz}$$

Using the expression (7) for the pdf in the above Eq. (11) and simplifying, we easily have

12 $$\begin{gathered} E\,\left( { - \ln f_{Z} (z)} \right) \hfill \\ \quad = \;\ln \left[ {\frac{{\left( {\alpha \; + \;1} \right)\,\left( {\beta \; + \;1} \right)}}{{\alpha^{2} \,\beta^{2} \,}}} \right]\; - \;E\,\left( {\ln \,\left( {\frac{{\left( {\left( {\alpha \; + \;2} \right)\,z\; + \;\beta } \right)\,\left( {\alpha \,z\; + \;\beta \; + \;2} \right)\; + \;2\,z}}{{\left( {\alpha \,z\; + \;\beta } \right)^{4} }}} \right)} \right). \hfill \\ \end{gathered}$$

Obviously, the expected value in Eq. (12) cannot be evaluated analytically in closed form and so requires some appropriate numerical quadrature formulas for computations. As such, we have computed the Shannon entropy of the distribution of the ratio $Z = \left| \frac{X}{Y} \right|$ numerically as provided in Table 1 for two sets of values: **(A)** for $\alpha \; = \;0.5$ and different values of $\beta$, and **(B)** for $\beta = 0.5$ and different values of $\alpha$.

**Table 1:** Shannon entropy of RV $Z = \left| \frac{X}{Y} \right|$

Set A			Set B		
Parameters		Shannon entropy:$E\,\left( { - \ln f_{Z} (z)} \right)$	Parameters		Shannon entropy:$E\,\left( { - \ln f_{Z} (z)} \right)$
$\alpha$	$\beta$		$\alpha$	$\beta$	
0.5	0.10	− 0.108726	0.10	0.5	3.595000
	0.20	0.691960	0.20		2.857871
	0.50	1.830920	0.50		1.830920
	1.00	2.731650	1.00		1.012020
	2.00	3.619645	2.00		0.180390
	3.00	4.118150	3.00		− 0.298705
	5.00	4.719200	5.00		− 0.885972

The behavior of entropy as given in Table 1 is evident for the two sets of values of the parameters $\alpha$ and $\beta$. It is observed from Set A that for fixed $\alpha \; = \;0.5$, the entropy is an increasing function of the parameters $\beta$. On the other hand, from Set B, we observe that, for fixed $\beta \; = \;0.5$, the entropy is a decreasing function of the parameters $\alpha$.

### Percentiles

The percentage points $z_{p}$ of the distribution of ratio $Z = \left| \frac{X}{Y} \right|$ by numerically solving the equations for the cdf in Theorem 1, that is, $F\,(z_{p} ) = \int\limits_{0}^{{z_{p} }} {f_{Z} (} u)\,du = p$ (say), for any $0 < p < 1$, or $z_{p} \; = \;F^{ - 1} \left( p \right)$, by taking different sets of values of the parameters. The percentage points $z_{p}$ associated with the cdf of $Z$ are computed by using Maple software for $\alpha \; = \;1$ and different values of $\beta$, and for $\beta = 0.5$ and different values of $\alpha$, respectively. These are provided in the Table 2. Similar percentage points $z_{p}$ can be obtained for other values of $\alpha$ and $\beta$. We believe that the percentile points will be useful for the researchers as mentioned in Sect. 1.

**Table 2:** Percentage points of $Z = \left| \frac{X}{Y} \right|$

		$$p$$					
$\alpha$	$\beta$	**75%**	**80%**	**85%**	**90%**	**95%**	99%
1	0.10	1.500	1.600	1.700	1.800	1.900	1.9900
	0.25	1.5024	1.6035	1.7122	1.8080	1.9023	1.9890
	0.50	1.4990	1.5959	1.7099	1.7909	1.8999	1.9880
	1.00	1.5055	1.5590	1.6991	1.8110	1.9100	1.9840
	2.00	1.5195	1.5985	1.7020	1.7999	1.8977	1.9999
	3.00	1.5105	1.5977	1.6990	1.80000	1.8999	1.9885
	5.00	1.4999	1.6100	1.7155	1.8145	1.9105	1.9777

		$$p$$					
$\alpha$	$\beta$	**75%**	**80%**	**85%**	**90%**	**95%**	99%
0.10	0.5	82.977	88.577	93.855	99.055	105.00	109.20
0.25		14.980	16.113	16.925	18.025	18.993	19.795
0.50		4.5540	4.825	5.1122	5.455	5.6795	5.999
1.00		1.4990	1.5990	1.7089	1.7909	1.8990	1.9899
2.00		0.5609	0.6050	0.6390	0.6795	0.71500	0.74500
3.00		0.3355	0.3590	0.3785	0.3990	0.4259	0.4399
5.00		0.1800	0.1910	0.2055	0.2160	0.2299	0.2390

## Characterizations

The characterization of a probability distributions plays an important role in probability and statistics. In order to apply a particular probability distribution to some real world data, it is very important to characterize it first subject to certain conditions. As pointed out by Nagaraja [26], “A characterization uses a certain distributional or statistical property of a statistic or statistics that uniquely determines the associated stochastic model”. There are various methods of characterizations to identify the distribution of a real data set, see for example Ahsanullah [3]. The general theories of characterization of distributions were discussed by Kagan et al. [15], followed by Galambos and Kotz [11], among others. One of the most important method of characterization is the method of truncated moments. We shall prove the characterization theorems by the left and right truncated conditional expectations of $Z^{k}$, by considering a product of reverse hazard rate and another function of the truncated point. We shall need the following assumption.

### Assumption 1

Suppose the random variable $Z$ is absolutely continuous with the cumulative distribution function $F(z)$ and the probability density function $f(z)$. We assume that $\omega \; = \;\inf \,\left\{ {\,z\,\,|\,\,F\,\left( z \right)\; > \;0\,} \right\}$, and $\delta \; = \;\sup \,\left\{ {\,z\;|\;F\,\left( z \right)\; < \;1\,} \right\}$. We also assume that $f(z)$ is a differentiable for all $z$, and $E\,\left( {Z^{k} } \right)$ exists, where $- 1\; < \;k\; < \;1$.

**Case 1:** We shall need the following lemma.

### Lemma 1

Suppose that a non-negative random variable ${\text{Z}}$ has an absolutely continuous (with respect to Lebesgue measure) cdf (${\text{F}}\left( Z \right)$) and pdf (${\text{f}}\left( z \right)$). Suppose the random variable ${\text{Z}}$ satisfies the Assumption 6.1 with $\omega \; = \;0$ and $\delta = \infty$. We assume that $f^{/} \left( z \right)$ exits for all ${\text{z}}$ and $0\; < \;E\,\left( {Z^{k} } \right)\; < \;\infty$, where $- 1\; < \;k\; < \;1$. Then, if

$$E(Z^{k} |Z \le \;z) = g\left( z \right)\,\tau \left( z \right),\;{\text{where}}\;\tau \left( z \right)\; = \;\frac{f\left( z \right)}{{F\left( z \right)}}\;{\text{for all}}\;z\; > \;0\;{\text{and}}\;g^{/} \left( z \right)\;{\text{exists for all}}\;z\; > \;0,$$

we have

$$f\left( z \right)\; = \;c\,e^{{\int {\frac{{z^{k} \; - \;g^{/} \left( z \right)}}{g\left( z \right)}} \,dz}} ,\;{\text{for all}}\;z\; > \;0,\;{\text{where}}\;c\;{\text{is a constant and}}\;c = \int_{0}^{\infty } {f\left( z \right)d} z.$$

### Proof

We have

$$E(Z^{k} |Z \le \;z) = \frac{{\int_{0}^{z} {u^{k} \,f\left( u \right)du} }}{F\left( z \right)}.$$.

Thus,

$$\int_{0}^{z} {u^{k} \,f\left( u \right)du} \; = \;g\left( z \right)\,f\left( z \right).$$

Differentiating both sides of the equation, we obtain

$$z^{k} \,f(z)\; = \;g^{/} (z)\,f(z)\; + \;g(z)\,f^{/} (z)\,,$$

or,

$$\frac{{f^{/} (z)}}{f(z)} = \frac{{z^{k} - g^{/} (z)\,}}{g(z)}$$

Integrating both sides of the above equation, we obtain.

$$f\left( z \right)\; = \;c\,e^{{\int {\frac{{z^{k} \; - \;g^{/} \left( z \right)}}{g\left( z \right)}} \,dz}} .$$.

for all $z\; > \;0$, where $c$ is a constant and $c = \int_{0}^{\infty } {f\left( z \right)d} z$. This completes the proof of Lemma 1.

### Theorem 4

Suppose that the random variable $Z$ satisfies the Assumption 1 with $\omega \; = \;0$ and $\delta = \infty$. Then $E(Z^{k} |Z \le \;z) = g\left( z \right)\,\frac{f\left( z \right)}{{F\left( z \right)}}$, where

13 $$g\,\left( z \right)\; = \;\frac{{I_{k} \left( z \right)\,}}{{\left( {\frac{{\alpha^{2} }}{\alpha \; + \;1}} \right)\,\,\left( {\frac{{\beta^{2} }}{\beta \; + \;1}} \right)\,\,\left[ {\frac{1}{{\left( {\alpha \,z\; + \;\beta } \right)^{2} }}\; + \;\frac{{2\,\left( {z\; + \;1} \right)}}{{\,\left( {\alpha \,z\; + \;\beta } \right)^{3} }}\; + \;\frac{6\,z}{{\,\left( {\alpha \,z\; + \;\beta } \right)^{4} }}} \right]}},$$

if and only if $Z$ has the pdf

$$f_{Z} \,\left( z \right)\; = \;\left( {\frac{{\alpha^{2} }}{\alpha \; + \;1}} \right)\,\,\left( {\frac{{\beta^{2} }}{\beta \; + \;1}} \right)\,\,\left[ {\frac{1}{{\left( {\alpha \,z\; + \;\beta } \right)^{2} }}\; + \;\frac{{2\,\left( {z\; + \;1} \right)}}{{\,\left( {\alpha \,z\; + \;\beta } \right)^{3} }}\; + \;\frac{6\,z}{{\,\left( {\alpha \,z\; + \;\beta } \right)^{4} }}} \right],$$

where $I_{k} \left( z \right) = \;\int_{0}^{z} {u^{k} } f\,\left( u \right)\,du$ denotes $kth$ incomplete moment of $Z$, (see Theorem A.1, Appendix II).

### Proof

Suppose that $E(Z^{k} |Z \le \;z) = g\left( z \right)\,\frac{f\left( z \right)}{{F\left( z \right)}}$. Then, since $E(Z^{k} |Z \le \;z)\; = \;\frac{{\int_{0}^{z} {u^{k} } \,f\,\left( u \right)\,du}}{{F_{\kern 1pt} \,\left( z \right)}}$, we have $g\,\left( z \right)\; = \;\frac{{\int_{0}^{z} {u^{k} } \,f\,\left( u \right)\,du}}{{f_{\kern 1pt} \,\left( z \right)}}$. Now, if the random variable ${\text{Z}}$ satisfies the Assumption 1 and has the distribution with the pdf (7), then we have

$$\begin{aligned} g\,\left( z \right)\; & = \;\frac{{\int_{0}^{z} {u^{k} } f\,\left( u \right)\,du}}{{f_{\kern 1pt} \,\left( z \right)}} \\ & = \;\frac{{I_{k} \left( z \right)\,}}{{\left( {\frac{{\alpha^{2} }}{\alpha \; + \;1}} \right)\,\,\left( {\frac{{\beta^{2} }}{\beta \; + \;1}} \right)\,\,\left[ {\frac{1}{{\left( {\alpha \,z\; + \;\beta } \right)^{2} }}\; + \;\frac{{2\,\left( {z\; + \;1} \right)}}{{\,\left( {\alpha \,z\; + \;\beta } \right)^{3} }}\; + \;\frac{6\,z}{{\,\left( {\alpha \,z\; + \;\beta } \right)^{4} }}} \right]}}, \\ \end{aligned}$$

where, as mentioned above, $I_{k} \left( z \right)\; = \;\int_{0}^{z} {u^{k} } f\,\left( u \right)\,du$ denotes $kth$ incomplete moment of $Z$.. Consequently, the proof of “if” part of Theorem 1 follows from Lemma 1.

Conversely, suppose that

$$g\,\left( z \right)\; = \;\frac{{I_{k} \left( z \right)\,}}{{\left( {\frac{{\alpha^{2} }}{\alpha \; + \;1}} \right)\,\,\left( {\frac{{\beta^{2} }}{\beta \; + \;1}} \right)\,\,\left[ {\frac{1}{{\left( {\alpha \,z\; + \;\beta } \right)^{2} }}\; + \;\frac{{2\,\left( {z\; + \;1} \right)}}{{\,\left( {\alpha \,z\; + \;\beta } \right)^{3} }}\; + \;\frac{6\,z}{{\,\left( {\alpha \,z\; + \;\beta } \right)^{4} }}} \right]}},$$

where $I_{k} \left( z \right)\; = \int_{0}^{z} {u^{k} } f\,\left( u \right)\,du$. Now, from Lemma 1, we have,

$$g\,\left( z \right)\; = \;\frac{{\int_{0}^{z} {u^{k} } f\,\left( u \right)\,du}}{{f_{\kern 1pt} \,\left( z \right)}},$$

or

$$\int_{0}^{z} {u^{k} } f\,\left( u \right)\,du = f(z)g(z).$$

Differentiating the above equation with respect to respect to $x$, we obtain

$$z^{k} \,f\,\left( z \right)\; = \;f^{/} \left( z \right)\,g(z)\; + \;f\left( z \right)\,g^{/} \left( z \right)$$

from which, using the pdf $f\,\left( z \right)$ and its first derivative $f^{/} \left( z \right)$, we haveor

$$g^{/} (z)\; = \;z^{k} \; - \;g\,\left( z \right)\,\frac{{\left[ {\frac{{ - 2\,\left( {\alpha \; - \;1} \right)}}{{\left( {\alpha \,z\; + \;\beta } \right)^{3} }}\; - \;\frac{6\,\alpha \,z}{{\left( {\alpha \,z\; + \;\beta } \right)^{4} }}\; - \;\frac{24\,\alpha \,z}{{\left( {\alpha \,z\; + \;\beta } \right)^{5} }}} \right]}}{{\,\left[ {\frac{1}{{\left( {\alpha \,z\; + \;\beta } \right)^{2} }}\; + \;\frac{{2\,\left( {z\; + \;1} \right)}}{{\,\left( {\alpha \,z\; + \;\beta } \right)^{3} }}\; + \;\frac{6\,z}{{\,\left( {\alpha \,z\; + \;\beta } \right)^{4} }}} \right]\,}},$$

14 $$\frac{{z^{k} \; - \;g^{/} (z)}}{g\,\left( z \right)}\; = \;\frac{{\left[ {\frac{{ - 2\,\left( {\alpha \; - \;1} \right)}}{{\left( {\alpha \,z\; + \;\beta } \right)^{3} }}\; - \;\frac{6\,\alpha \,z}{{\left( {\alpha \,z\; + \;\beta } \right)^{4} }}\; - \;\frac{24\,\alpha \,z}{{\left( {\alpha \,z\; + \;\beta } \right)^{5} }}} \right]}}{{\,\left[ {\frac{1}{{\left( {\alpha \,z\; + \;\beta } \right)^{2} }}\; + \;\frac{{2\,\left( {z\; + \;1} \right)}}{{\,\left( {\alpha \,z\; + \;\beta } \right)^{3} }}\; + \;\frac{6\,z}{{\,\left( {\alpha \,z\; + \;\beta } \right)^{4} }}} \right]\,}}.$$

Since, by Lemma 1, we have

15 $$\frac{{f^{/} (z)}}{f(z)} = \frac{{z^{k} - g^{/} (z)\,}}{g(z)},$$

see Shakil et al. [33]

Therefore, from (14) and (15), it follows that

16 $$\frac{{f^{/} \left( z \right)}}{f\left( z \right)}\; = \;\frac{{\left[ {\frac{{ - 2\,\left( {\alpha \; - \;1} \right)}}{{\left( {\alpha \,z\; + \;\beta } \right)^{3} }}\; - \;\frac{6\,\alpha \,z}{{\left( {\alpha \,z\; + \;\beta } \right)^{4} }}\; - \;\frac{24\,\alpha \,z}{{\left( {\alpha \,z\; + \;\beta } \right)^{5} }}} \right]}}{{\,\left[ {\frac{1}{{\left( {\alpha \,z\; + \;\beta } \right)^{2} }}\; + \;\frac{{2\,\left( {z\; + \;1} \right)}}{{\,\left( {\alpha \,z\; + \;\beta } \right)^{3} }}\; + \;\frac{6\,z}{{\,\left( {\alpha \,z\; + \;\beta } \right)^{4} }}} \right]\,}}.$$

Now, integrating Eq. (16) with respect to $z$ and simplifying, we haveor

$$\ln \left( {f\left( z \right)} \right)\; = \;\ln \left( {c\,\,\left[ {\frac{1}{{\left( {\alpha \,z\; + \;\beta } \right)^{2} }}\; + \;\frac{{2\,\left( {z\; + \;1} \right)}}{{\,\left( {\alpha \,z\; + \;\beta } \right)^{3} }}\; + \;\frac{6\,z}{{\,\left( {\alpha \,z\; + \;\beta } \right)^{4} }}} \right]} \right),$$

17 $$f\left( z \right)\; = \;c\,\left( {\,\left[ {\frac{1}{{\left( {\alpha \,z\; + \;\beta } \right)^{2} }}\; + \;\frac{{2\,\left( {z\; + \;1} \right)}}{{\,\left( {\alpha \,z\; + \;\beta } \right)^{3} }}\; + \;\frac{6\,z}{{\,\left( {\alpha \,z\; + \;\beta } \right)^{4} }}} \right]} \right),$$

where $c$ is the normalizing constant to be determined. Integrating Eq. (17) with respect to $z$ from $z\; = \;0$ to $x\; = \;\infty$, and using the condition $\int_{0}^{\infty } {f\,\left( z \right)} dz = 1$, we obtain

18 $$\frac{1}{c}\; = \;\int\limits_{0}^{\infty } {\left( {\,\left[ {\frac{1}{{\left( {\alpha \,z\; + \;\beta } \right)^{2} }}\; + \;\frac{{2\,\left( {z\; + \;1} \right)}}{{\,\left( {\alpha \,z\; + \;\beta } \right)^{3} }}\; + \;\frac{6\,z}{{\,\left( {\alpha \,z\; + \;\beta } \right)^{4} }}} \right]} \right)} \,\,dz.$$

Now, substituting $\alpha \,z\; + \;\beta \; = \;u$ in (16), then integrating it with respect to $u$ from $u\; = \;\beta$ to $t\; = \;\infty$, and simplifying, it is easily seen that.

$$\frac{1}{c}\; = \;\;\left( {\frac{\alpha \; + \;1}{{\alpha^{2} }}} \right)\,\,\left( {\frac{\beta \; + \;1}{{\beta^{2} }}} \right),$$

This completes the proof of Theorem 1.

**Case 2:** We shall need the following lemma.

### Lemma 2

Suppose that a non-negative random variable ${\text{Z}}$ has an absolutely continuous (with respect to Lebesgue measure) cdf (${\text{F}}\left( Z \right)$) and pdf (${\text{f}}\left( z \right)$). Suppose the random variable ${\text{Z}}$ satisfies the Assumption 1 with $\omega \; = \;0$ and $\delta = \infty$. We assume that $f^{/} \left( z \right)$ exits for all ${\text{z}}$ and $0\; < \;E\,\left( {Z^{k} } \right)\; < \;\infty$, where $- 1\; < \;k\; < \;1$. Then, if $E(Z^{k} |Z\; \ge \;z) = h\left( z \right)\,r\left( z \right)$, where $r\left( z \right)\; = \;\frac{f\left( z \right)}{{1\; - \;F\left( z \right)}}$ for all $z\; > \;0$ and $h\,\left( z \right)$ is a continuous differentiable function of $z$ with the condition that $\,\int_{z}^{\infty } {\,\frac{{u^{k} \; + \;\left[ {h\,\left( u \right)} \right]^{/} }}{h\,\left( u \right)}\,du}$ is finite for $z > 0$, then $f\left( z \right)\; = \;c\,e^{{ - \,\int_{z}^{\infty } {\,\frac{{u^{k} \; + \;\left[ {h\,\left( u \right)} \right]^{/} }}{h\,\left( u \right)}\,du} }}$, where $c$ is a constant determined by the condition $\int_{0}^{\infty } {f(z)} dz\; = \;1$.

### Proof

The proof is similar to Lemma 1**.**

### Theorem 5

Suppose that the random variable ${\text{Z}}$ satisfies the Assumption 1 with $\omega \; = \;0$ and $\delta = \infty$. Then $E(Z^{k} |Z \ge \;z) = h\left( z \right)\,\frac{f\left( z \right)}{{1\; - \;F\left( z \right)}}$, where $h\left( z \right)\; = \;\frac{{\left( {E\left( {Z^{k} } \right)\; - \;g\left( z \right)\,f\left( z \right)} \right)}}{f\left( z \right)}$, $E\left( {Z^{k} } \right)$ is given by Eq. (9), and $g\left( z \right)$ is given by Eq. (13), if and only if ${\text{Z}}$ has the pdf

$$f_{Z} \,\left( z \right)\; = \;\left( {\frac{{\alpha^{2} }}{\alpha \; + \;1}} \right)\,\,\left( {\frac{{\beta^{2} }}{\beta \; + \;1}} \right)\,\,\left[ {\frac{1}{{\left( {\alpha \,z\; + \;\beta } \right)^{2} }}\; + \;\frac{{2\,\left( {z\; + \;1} \right)}}{{\,\left( {\alpha \,z\; + \;\beta } \right)^{3} }}\; + \;\frac{6\,z}{{\,\left( {\alpha \,z\; + \;\beta } \right)^{4} }}} \right].$$

### Proof

Proceeding in the same way as in Theorem 4, following similar arguments, the proof of Theorem 5 easily follows using Lemma 2.

## Estimation

In this section, we provide the estimation of the parameters of the distribution of ratio $Z = \left| \frac{X}{Y} \right|$ by the method of maximum likelihood (MLE). Given a sample $\left\{ {\,z_{i} \,} \right\}$, $i\; = \;1,\;2,\;3,\; \ldots ,\;n$, from a population with the distribution of ratio $Z = \left| \frac{X}{Y} \right|$, the likelihood function is given by

$$\begin{aligned} L\,\left( {\alpha ,\;\beta } \right) & \; = \;\prod\limits_{i\; = \;1}^{n} {\,f\,\,\left( {\,z_{i} ,\;\alpha ,\;\beta } \right)} \\ & = \;\prod\limits_{i\; = \;1}^{n} \, \left( {\frac{{\alpha^{2} }}{\alpha \; + \;1}} \right)\,\,\left( {\frac{{\beta^{2} }}{\beta \; + \;1}} \right)\,\,\left[ {\frac{1}{{\left( {\alpha \,z_{i} \; + \;\beta } \right)^{2} }}\; + \;\frac{{2\,\left( {z_{i} \; + \;1} \right)}}{{\,\left( {\alpha \,z_{i} \; + \;\beta } \right)^{3} }}\; + \;\frac{{6\,z_{i} }}{{\,\left( {\alpha \,z_{i} \; + \;\beta } \right)^{4} }}} \right] \\ & = \;\left( {\frac{{\alpha^{2} }}{\alpha \; + \;1}} \right)^{n} \,\,\left( {\frac{{\beta^{2} }}{\beta \; + \;1}} \right)^{n} \,\,\prod\limits_{i\; = \;1}^{n} \, \,\left[ {\frac{1}{{\left( {\alpha \,z_{i} \; + \;\beta } \right)^{2} }}\; + \;\frac{{2\,\left( {z_{i} \; + \;1} \right)}}{{\,\left( {\alpha \,z_{i} \; + \;\beta } \right)^{3} }}\; + \;\frac{{6\,z_{i} }}{{\,\left( {\alpha \,z_{i} \; + \;\beta } \right)^{4} }}} \right] \\ \end{aligned}$$

The objective of the likelihood function approach is to determine those values of the parameters that maximize the function $L$. Then, taking the natural logarithm, the log-likelihood function is given by

$$\begin{aligned} R\; & = \;\ln \,\left( {\,L\,} \right) \\ & = \;n\,\left( {2\ln \alpha \; - \;\ln \left( {\alpha \; + \;1} \right)} \right)\; + \;n\,\left( {2\ln \beta \; - \;\ln \left( {\beta \; + \;1} \right)} \right) \\ & \;\;\, + \;\sum\limits_{i\; = \;1}^{n} {\ln \left[ {\frac{1}{{\left( {\alpha \,z_{i} \; + \;\beta } \right)^{2} }}\; + \;\frac{{2\,\left( {z_{i} \; + \;1} \right)}}{{\,\left( {\alpha \,z_{i} \; + \;\beta } \right)^{3} }}\; + \;\frac{{6\,z_{i} }}{{\,\left( {\alpha \,z_{i} \; + \;\beta } \right)^{4} }}} \right]} . \\ \end{aligned}$$

Thus, the maximum likelihood estimates (MLE) of the parameters $\alpha$ and $\beta$ are obtained by setting $\frac{\partial \,R}{{\partial \,\alpha }}\; = \;0$, $\frac{\partial \,R}{{\partial \,\beta }}\; = \;0$, from which we obtain the following system of maximum likelihood equations:

19 $$\frac{\partial R}{\partial \alpha }\hspace{0.33em}=\hspace{0.33em}\frac{n\left(\alpha \hspace{0.33em}+2\right)}{\alpha \left(\alpha \hspace{0.33em}+\hspace{0.33em}1\right)}\hspace{0.33em}-\hspace{0.33em}{\sum }_{i\hspace{0.33em}=\hspace{0.33em}1}^{n}\frac{\frac{2{z}_{i}}{{\left(\alpha {z}_{i}\hspace{0.33em}+\hspace{0.33em}\beta \right)}^{3}}\hspace{0.33em}+\hspace{0.33em}\frac{6{z}_{i}\left({z}_{i}\hspace{0.33em}+\hspace{0.33em}1\right)}{{\left(\alpha {z}_{i}\hspace{0.33em}+\hspace{0.33em}\beta \right)}^{4}}\hspace{0.33em}+\hspace{0.33em}\frac{24{{z}_{i}}^{2}}{{\left(\alpha {z}_{i}\hspace{0.33em}+\hspace{0.33em}\beta \right)}^{5}}\hspace{0.33em}}{\left[\frac{1}{{\left(\alpha {z}_{i}\hspace{0.33em}+\hspace{0.33em}\beta \right)}^{2}}\hspace{0.33em}+\hspace{0.33em}\frac{2\left({z}_{i}\hspace{0.33em}+\hspace{0.33em}1\right)}{{\left(\alpha {z}_{i}\hspace{0.33em}+\hspace{0.33em}\beta \right)}^{3}}\hspace{0.33em}+\hspace{0.33em}\frac{6{z}_{i}}{{\left(\alpha {z}_{i}\hspace{0.33em}+\hspace{0.33em}\beta \right)}^{4}}\right]}\hspace{0.33em}=\hspace{0.33em}0,$$

20 $$\frac{\partial R}{\partial \beta }\hspace{0.33em}=\hspace{0.33em}\frac{n\left(\beta \hspace{0.33em}+2\right)}{\beta \left(\beta +\hspace{0.33em}1\right)}\hspace{0.33em}-\hspace{0.33em}{\sum }_{i\hspace{0.33em}=\hspace{0.33em}1}^{n}\frac{\frac{2}{{\left(\alpha {z}_{i}\hspace{0.33em}+\hspace{0.33em}\beta \right)}^{3}}\hspace{0.33em}+\hspace{0.33em}\frac{6\left({z}_{i}\hspace{0.33em}+\hspace{0.33em}1\right)}{{\left(\alpha {z}_{i}\hspace{0.33em}+\hspace{0.33em}\beta \right)}^{4}}\hspace{0.33em}+\hspace{0.33em}\frac{24{z}_{i}}{{\left(\alpha {z}_{i}\hspace{0.33em}+\hspace{0.33em}\beta \right)}^{5}}\hspace{0.33em}}{\left[\frac{1}{{\left(\alpha {z}_{i}\hspace{0.33em}+\hspace{0.33em}\beta \right)}^{2}}\hspace{0.33em}+\hspace{0.33em}\frac{2\left({z}_{i}\hspace{0.33em}+\hspace{0.33em}1\right)}{{\left(\alpha {z}_{i}\hspace{0.33em}+\hspace{0.33em}\beta \right)}^{3}}\hspace{0.33em}+\hspace{0.33em}\frac{6{z}_{i}}{{\left(\alpha {z}_{i}\hspace{0.33em}+\hspace{0.33em}\beta \right)}^{4}}\right]}\hspace{0.33em}=0.$$

Solving the above system of maximum likelihood Eqs. (19) and (20) by applying the Newton–Raphson’s iteration method and using the computer package such as Maple, or R or MathCAD14, or other software, the maximum likelihood estimates (MLE) of the parameters $\alpha$ and $\beta$ can be obtained.

## Applications

We demonstrate the applicability of our proposed EDTPL by considering two different data sets, namely, Bladder Cancer and COVID-19. The goodness-of-fit tests of our proposed EDTPL distribution is provided by comparing it with the fitting of some well-known right-skewed variations of Lindley distribution, namely; the LD Lindley [17], the NGLD Elbatal et al. [9], the NQLD (Shanker and Amanuel (34)), and ATPLD Shanker et al. [36], to the above said data sets. The parameters are estimated by using the maximum likelihood method, and for comparison we use − LL, K-S test, AIC, CAIC, BIC and HQIC. For details on these, the interested readers are also referred to Emiliano et al. [10]. All calculations for these criterions are executed by the computational software “*MATHEMATICA* 11.0”. The smaller values of these criteria for a distribution imply that the distribution fits better to the data.

### Example 1

 Application to Data for Bladder Cancer Patients

We consider an uncensored data set corresponding to remission times (in months) of a random sample of 128 bladder cancer patients (Lee and Wang (19)) as presented in Table 3.

**Table 3:** Data for bladder cancer patients

The remission times (in months) of bladder cancer patients	
0.08 2.09 3.48 4.87 6.94 8.66 13.11 23.63 0.2 2.23 0.26 0.31 0.73 0.52 4.98 6.97 9.02 13.29 0.4 2.26 3.57 5.06 7.09 11.98 4.51 2.07 0.22 13.8 25.74 0.5 2.46 3.64 5.09 7.26 9.47 14.24 19.13 6.54 3.36 0.82 0.51 2.54 3.7 5.17 7.28 9.74 14.76 26.31 0.81 1.76 8.53 6.93 0.62 3.82 5.32 7.32 10.06 14.77 32.15 2.64 3.88 5.32 3.25 12.03 8.65 0.39 10.34 14.83 34.26 0.9 2.69 4.18 5.34 7.59 10.66 4.5 20.28 12.63 0.96 36.66 1.05 2.69 4.23 5.41 7.62 10.75 16.62 43.01 6.25 2.02 22.69 0.19 2.75 4.26 5.41 7.63 17.12 46.12 1.26 2.83 4.33 8.37 3.36 5.49 0.66 11.25 17.14 79.05 1.35 2.87 5.62 7.87 11.64 17.36 12.02 6.76 0.4 3.02 4.34 5.71 7.93 11.79 18.1 1.46 4.4 5.85 2.02 12.07	

We have tested the normality of the data by Ryan-Joiner Test (Similar to Shapiro–Wilk Test), which is provided in Table 4.

**Table 4:** Ryan-Joiner normality assessment

Normality assessment	
Ryan-Joiner test Test statistic, Rp: 0.8151 Critical value for 0.05 significance level: 0.9887 Critical value for 0.01 significance level: 0.9842 Reject normality with a 0.05 significance level Reject normality with a 0.01 significance level Possible outliers Number of data values below Q1 by more than 1.5 IQR: 0 Number of data values above Q3 by more than 1.5 IQR: 9	

From Table 4 of Ryan-Joiner Test of Normality Assessment, it is obvious that the shape of the data for bladder cancer patients is skewed to the right with a heavy-tailed distribution and is leptokurtic. This is also obvious from the skewness and kurtosis of the data which are computed as 3.4175 and 16.7810, respectively. Furthermore, since fitting of a probability distribution to the data for bladder cancer patients may be helpful in predicting the probability or forecasting the frequency of occurrence of the bladder cancer of patients, this suggests that y, the bladder cancer of patients, could possibly be modeled by some skewed distributions. Since our data are skewed in nature, we fitted our proposed ratio distribution to this data and compared it with the above-stated variations of Lindley distribution. The measures of goodness-of-fit including the AIC, CAIC, BIC, HQIC and K-S statistics are computed to compare the fitted models, which are provided in Table 5. In general, the distribution with smaller values of these statistics better fits to the data.

**Table 5:** The estimators of parameters for bladder cancer patients

Model	Parameters	−LL	AIC	CAIC	BIC	HQIC	K-S statistics
EDTPL	$\widehat{\alpha }=$ 0.06458 $\widehat{\beta }=$ 0.30005	408.62	821.23	821.326	826.934	823.547	0.0557
LD	$\widehat{\theta }=$ 0.196	419.52	841.040	841.072	843.892	842.199	0.0740
NGLD	$\widehat{\theta }=$ 0.180 $\widehat{\alpha }=$ 4.679 $\widehat{\beta }=$ 1.324	412.75	831.501	831.694	840.057	834.97	0.1160
NQLD	$\widehat{\alpha }=0.949$, $\widehat{\theta }$=0.225	427.54	859.087	859.183	864.791	861.405	0.9154
ATPLD	$\widehat{\alpha }=$ 0.9488 $\widehat{\beta }=$ 0.51 $\widehat{\theta }=$ 0.2246	414.99	835.986	836.18	844.542	839.463	0.9218

**Fig. 6:**
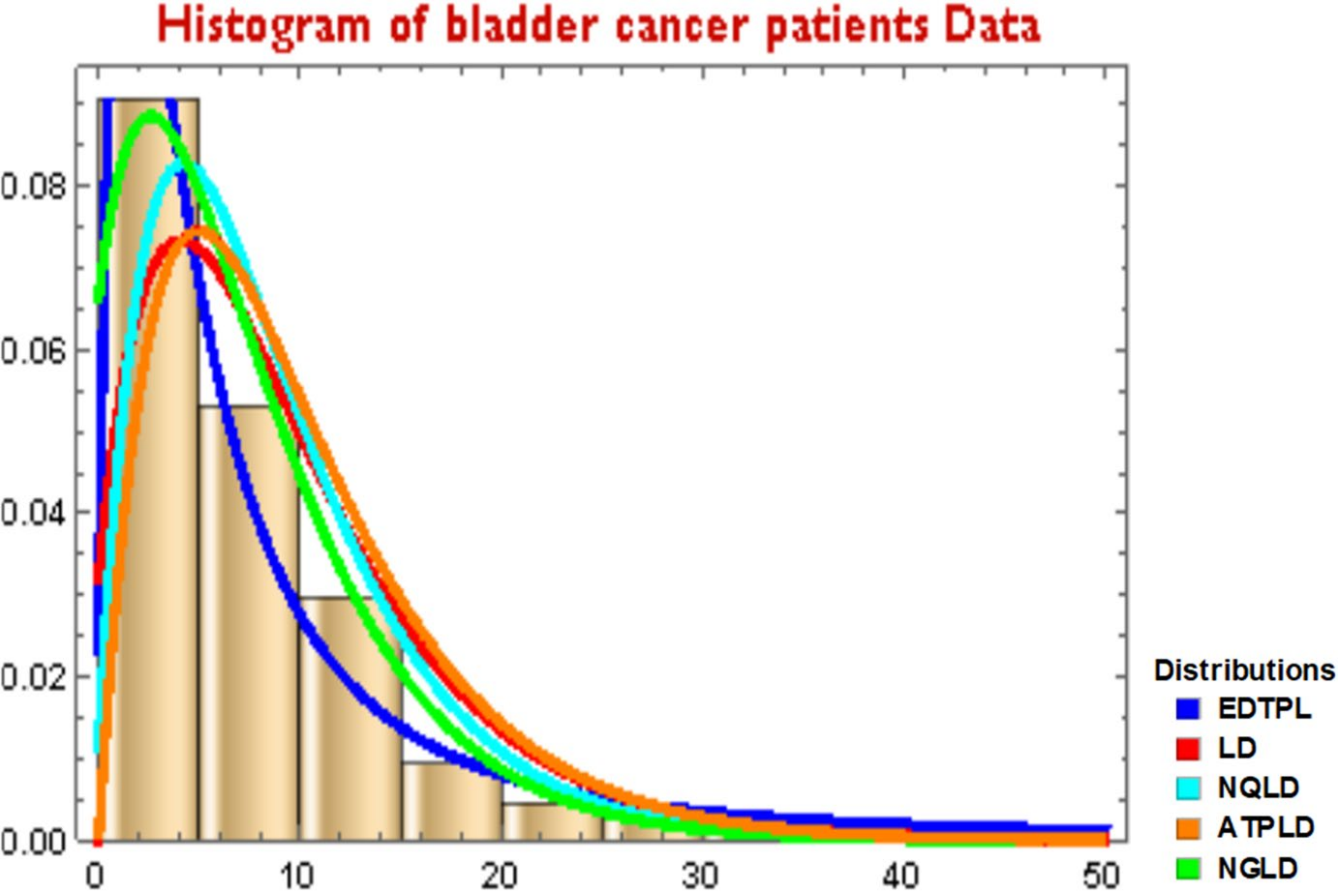
Fitted densities for cancer data (Table 8)

*Data Analysis*: It is observed from the above results in Table 5 that our proposed ratio distribution (EDTPL) has smaller values of the AIC, CAIC, BIC, HQIC and K-S statistics as compared to the distributions, namely; the LD, the NGLD, the NQLD, and ATPLD. Therefore, we conclude that our proposed ratio distribution (EDTPL) fits better to the data for bladder cancer patients than the rest of the distributions considered here. For the estimated parameters, the pdfs of these distributions have been superimposed on the histogram of the said cancer data set as provided in Fig. 6.

### Example 2

Application to COVID-19 Data of Italy:

The second data represents COVID-19 mortality rates data of Italy for 59 days that is recorded from 27 February to 27 April 2020. The data are provided in Table 6 as follows:

**Table 6:** COVID-19 data of Italy

COVID-19 mortality rates data of Italy for 59 days	
4.571 7.201 3.606 8.479 11.410 8.961 10.919 10.908 6.503 18.474 11.010 17.337 16.561 13.226 15.137 8.697 15.787 13.333 11.822 14.242 11.273 14.330 16.046 11.950 10.282 11.775 10.138 9.037 12.396 10.644 8.646 8.905 8.906 7.407 7.445 7.214 6.194 4.640 5.452 5.073 4.416 4.859 4.408 4.639 3.148 4.040 4.253 4.011 3.564 3.827 3.134 2.780 2.881 3.341 2.686 2.814 2.508 2.450 1.518	

We have tested the normality of the data by Ryan-Joiner Test (Similar to Shapiro–Wilk Test), which is provided in Table 7.

**Table 7:** Ryan-Joiner normality assessment

Normality assessment	
Ryan-Joiner test Test statistic, Rp: 0.9723 Critical value for 0.05 significance level: 0.9796 Critical value for 0.01 significance level: 0.9706 Reject normality with a 0.05 significance level Fail to reject normality with a 0.01 significance level Possible Outliers Number of data values below Q1 by more than 1.5 IQR: 0 Number of data values above Q3 by more than 1.5 IQR: 0	

The skewness and kurtosis of the data are computed as 0.464258 and − 0.841489, respectively. It is obvious from these and the Table 7 of Ryan-Joiner Test of Normality Assessment that the shape of the data for COVID-19 mortality rates is skewed to the right with a light-tailed distribution and lack of outliers, and is platykurtic. Furthermore, since fitting of a probability distribution to the data for COVID-19 mortality rates may be helpful in predicting the probability or forecasting the frequency of occurrence of the COVID-19 mortality rates, this suggests that y, the COVID-19 mortality rates, could possibly be modeled by some skewed distributions. Since our data are skewed in nature, we fitted our proposed ratio distribution (EDTPL) to this data and compared it with those of the above-mentioned right-skewed variations of Lindley distribution; the LD, the NGLD, the NQLD and ATPLD. The measures of goodness-of-fit including the AIC, CAIC, BIC, HQIC and K-S statistics are computed to compare the fitted models, which are provided in Table 8. In general, the distribution with the smaller values of these statistics better fits to the data.

**Table 8:** The estimators of parameters for COVID-19 data of Italy

Model	Parameters	−LL	AIC	CAIC	BIC	HQIC	K-S statistics
EDTPL	$\widehat{\alpha }=$ 0.05308 $\widehat{\beta }=$ 0.36864	187.563	379.128	379.342	383.283	380.75	0.0383
LD	$\widehat{\theta }=$ 0.563482	242.254	486.508	486.578	488.586	487.319	0.5240
NGLD	$\widehat{\theta }=$ 0.23099 $\widehat{\alpha }=$ 0.6496 $\widehat{\beta }=$ 1.1728	193.574	393.149	393.586	399.382	395.582	0.2529
NQLD	$\widehat{\alpha }=$ 1.03321 $\widehat{\theta }$ = 0.392515	190.0392	384.079	384.293	388.234	385.701	0.1266
ATPLD	$\widehat{\alpha }$= 0.127468 $,$ $\widehat{\beta }=-$ 0.12298, $\widehat{\theta }=$ 0.563482	187.918	378.636	380.072	384.869	381.069	0.09938

**Fig. 7:**
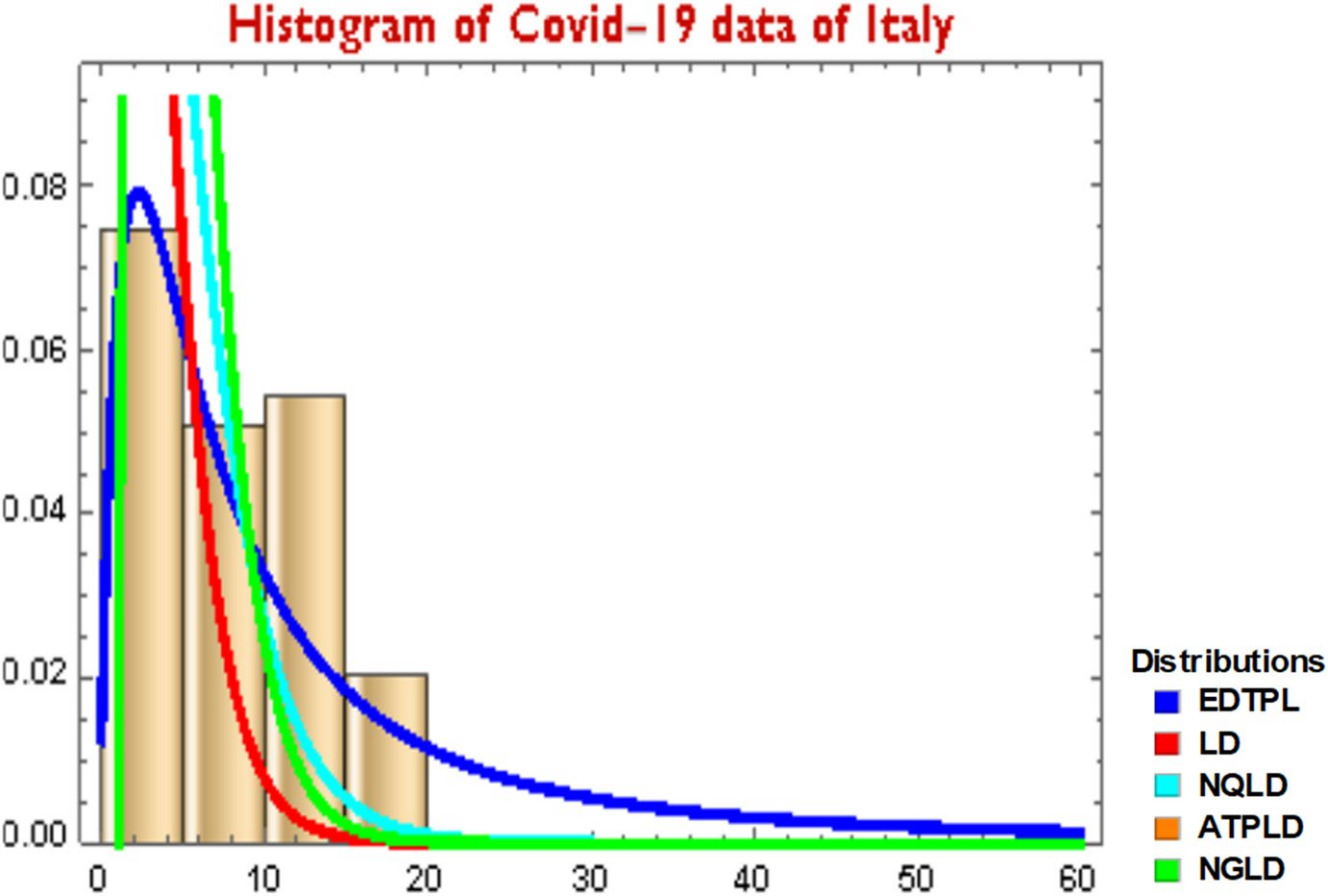
Fitted densities for COVID-19 data (Table 4)

*Data Analysis*: It is observed from the results given above in Table 8 that our proposed exact distribution (EDTPL) has smaller values of the AIC, CAIC, BIC, HQIC and K-S statistics as compared to the compared distributions. Therefore, we conclude that our proposed exact distribution (EDTPL) fits better to the COVID-19 mortality rates data of Italy for 59 days (recorded from 27 February to 27 April 2020) than the LD, the NGLD, the NQLD and ATPLD. For the estimated parameters, the pdfs of these distributions have been superimposed on the histogram of the said COVID-19 mortality rates data set as provided in Fig. 7.

## Concluding Remarks

This paper has derived the exact distribution of the ratio of two independent Lindley random variables $X$ and $Y$. The expressions for the associated cdf, pdf, moment and entropy of the ratio of two variables are given. The plots for the pdf, cdf and moment are provided. The percentile points, characterizations, parameter estimation and some applications are also given. We hope that the topic is practically significant and the proposed model may attract wider applications in many areas of real-world data sets. Furthermore, we hope the findings of the paper will be useful for the practitioners in various fields of applied sciences as mentioned in Sect. 1.

## Data Availability

Not applicable.

## Appendix I

### Descriptions of Some Special Functions and Lemmas

These are given below. For details, see, for example, Abramowitz and Stegun [1], Prudnikov et al. [30], and Gradshteyn and Ryzhik [13], among others. The series

$${}_{p}F_{q} \left( {\alpha_{1} ,\;\alpha_{2} ,\, \cdots \,,\,\alpha_{p} ;\;\beta_{1} ,\;\beta_{2} ,\, \cdots \,,\,\beta_{q} ;\;z} \right) = \sum\limits_{k\, = \,0}^{\infty } {\left\{ {\frac{{\left( {\alpha_{1} } \right)_{k} \;\left( {\alpha_{2} } \right)_{k} \, \cdots \,\,\left( {\alpha_{p} } \right)_{k} \;}}{{\left( {\beta_{1} } \right)_{k} \;\left( {\beta_{2} } \right)_{k} \, \cdots \,\,\left( {\beta_{q} } \right)_{k} }}\frac{{z^{k} }}{k!}} \right\}}$$,

is called a generalized hypergeometric series of order $\,(p,\;q)$, where $(\alpha )_{k}$ and $(\beta )_{k}$ represent Pochhammer symbols. For $p = 2$ and $q = 1$, we have generalized hypergeometric function ${}_{2}F_{1}$ of order $\,(2,\;1)$, given by ${}_{2}F_{1} \,\left( {\alpha \,,\;\beta \,;\;\gamma ;\;z} \right) \equiv F\,\left( {\alpha \,,\;\beta \,;\;\gamma ;\;z} \right)\; \equiv F\,\left( {\beta \,,\;\alpha \,;\;\gamma ;\;z} \right) = \sum\limits_{k\, = \,0}^{\infty } {\left\{ {\frac{{(\alpha )_{k} (\beta )_{k} \,z^{k} }}{{(\gamma )_{k} \,k!\,\,}}} \right\}}$. Also, we have $F\,\left( {\alpha \,,\;\beta \,;\;\gamma ;\;z} \right) = (1 - z)^{ - \,\beta } F\,\left( {\beta \,,\;\gamma - \alpha \,;\;\gamma ;\;\frac{z}{z - 1}} \right)$. The integrals $\Gamma (\alpha ) = \int\limits_{0}^{\infty } {t^{\alpha \, - \,1} e^{ - t} \,dt}$, and $\gamma \,\left( {\alpha ,\,\,x} \right) = \int\limits_{0}^{x} {t^{\alpha \, - \,1} e^{ - t} \,dt,\;\alpha > 0}$ are called (complete) gamma and incomplete gamma functions respectively, whereas the integral $\Gamma (\alpha ,\;x) = \int\limits_{x}^{\infty } {t^{\alpha \, - \,1} e^{ - t} \,dt} ,\;\alpha > 0$ is called the complementary incomplete gamma function. For negative values, gamma function can be defined as $\Gamma \left( { - n + \frac{1}{2}} \right) = \frac{{( - 1)^{n} \,2^{n} \,\sqrt \pi }}{1.3.5.\,\, \ldots \,(2n - 1)\,}$, where $n \ge 0$ is an integer, (see, for example, Abramowitz and Stegun [1], and Gradshteyn and Ryzhik [14], among others). The function defined by $B\,\left( {p,\;q} \right) = \int\limits_{0}^{1} {t^{p} \,(1 - t)^{q\, - \,1} dt} = \frac{\Gamma (p)\,\Gamma (q)}{{\Gamma (p + q)}},\;p > 0,\;q > 0,$ is known as beta function (or Euler’s function of the first kind). The error function is defined by $erf(x) = \frac{2}{\sqrt \pi }\int\limits_{0}^{x} {e^{{ - u^{2} }} du}$, whereas the complementary error, $erfc\left( x \right)$, is defined as $erfc(x) = \frac{2}{\sqrt \pi }\int\limits_{x}^{\infty } {e^{{ - u^{2} }} du} = 1 - erf(x)$.

The following six Lemmas will also be needed to complete the derivations.

#### Lemma A.1

Gradshteyn and Ryzhik [13], [5], Eq. (3.381.4), Page 317).

For $${\text{Re}} \,\,\left( \mu \right) > 0$$, and $${\text{Re}} \,\left( \nu \right) > 0$$,

$$\quad \;\int\limits_{0}^{\infty } {t^{\nu \, - \,1} \,e^{ - \,\mu \,\,t} } \,dt = \frac{1}{{\mu^{\nu } }}\Gamma (\nu )$$.

#### Lemma A.2

Gradshteyn and Ryzhik [13], [5], Eq. (3.194.3), Page 285).

$\quad \;\int\limits_{0}^{\infty } {\frac{{x^{\mu \,\, - \,\,1} }}{{\left( {1 + \beta \,x} \right)^{\nu } }}} \,\,\,dx = \;\beta^{ - \,\mu } \,\,B\,\left( {\mu \,,\;\nu - \mu } \right)\,,\quad {\text{where}}\quad \left[ {\left| {\,\arg \,(\beta )\,} \right| < \pi ,\quad \;{\text{Re}} \,\left( \nu \right) > {\text{Re}} \,\left( \mu \right) > 0} \right]$ where $B\,\left( . \right)$ denotes beta function (or Euler’s function of the first kind), (see definition above).

#### Lemma A.3

Gradshteyn and Ryzhik [13], [5], Eq. (3.194.1), Page 284).

$$\;\int\limits_{0}^{u} {\frac{{x^{\mu \,\, - \,\,1} }}{{\left( {1 + \beta \,x} \right)^{\nu } }}} \,\,\,dx = \;\frac{{u^{\mu } }}{\mu }\,{}_{2}F_{1} \,\left( {\nu ,\;\mu ;\;1\; + \;\mu ;\; - \,\beta \,u} \right)\quad {\text{where}}\quad \left[ {\left| {\,\arg \,(1\; + \;\beta \,u)\,} \right| < \pi ,\quad {\text{Re}} \,\left( \mu \right) > 0} \right]$$

where ${}_{2}F_{1} \,\left( . \right)$ denotes the generalized hypergeometric function of order $\,(2,\;1)$, (see definition above).

## Appendix II

### $kth$ Incomplete Moment of RV $Z = \left| \frac{X}{Y} \right|$

It is derived as follows.

#### Theorem A.1

If $Z$ is a random variable with pdf given by (7), then its $kth$ incomplete moment is given by

$$\begin{gathered} I_{k} \left( z \right) = = \;\left( {\frac{{\alpha^{2} }}{\alpha \; + \;1}} \right)\,\,\left( {\frac{{\beta^{2} }}{\beta \; + \;1}} \right)\,\left[ {\frac{{z^{k\; + \;1} }}{{\beta^{2} \,\left( {k\; + \;1} \right)}}\,{}_{2}F_{1} \,\left( {2,\;k\; + \;1;\;k\; + \;2;\; - \,\frac{\alpha }{\beta }\,z} \right)\;} \right. \hfill \\ + \;\frac{{2\,z^{k\; + \;2} }}{{\beta^{3} \,\left( {k\; + \;2} \right)}}\,{}_{2}F_{1} \,\left( {3,\;k\; + \;2;\;k\; + \;3;\; - \,\frac{\alpha }{\beta }\,z} \right) + \;\frac{{2\,z^{k\; + \;1} }}{{\beta^{3} \,\left( {k\; + \;1} \right)}}\,{}_{2}F_{1} \,\left( {3,\;k\; + \;1;\;k\; + \;2;\; - \,\frac{\alpha }{\beta }\,z} \right) \hfill \\ \left. { + \;\frac{{6\,z^{k\; + \;2} }}{{\beta^{4} \,\left( {k\; + \;2} \right)}}\,{}_{2}F_{1} \,\left( {4,\;k\; + \;2;\;k\; + \;3;\; - \,\frac{\alpha }{\beta }\,z} \right)} \right] \hfill \\ \end{gathered}$$

where $- 1\; < \;k\; < \;1$ and ${}_{2}F_{1} \,\left( . \right)$ denotes the generalized hypergeometric function of order $\,(2,\;1)$.

#### Proof

We have

21 $$\begin{aligned} I_{k} \left( z \right)\; & = \;\int\limits_{0}^{z} {u^{k} \,f\,\left( u \right)\,du} \\ & = \;\int\limits_{0}^{z} {u^{k} } \left( {\frac{{\alpha^{2} }}{\alpha \; + \;1}} \right)\,\,\left( {\frac{{\beta^{2} }}{\beta \; + \;1}} \right)\,\,\left[ {\frac{1}{{\left( {\alpha \,u\; + \;\beta } \right)^{2} }}\; + \;\frac{{2\,\left( {u\; + \;1} \right)}}{{\,\left( {\alpha \,u\; + \;\beta } \right)^{3} }}\; + \;\frac{6\,u}{{\,\left( {\alpha u\; + \;\beta } \right)^{4} }}} \right]\;du \\ \end{aligned}$$

Using Lemma A.3, integrating and simplifying, the Theorem A.1 easily follows, provided $- 1\; < \;k\; < \;1$.

## Abbreviations

Cdf: Cumulative distribution function
pdf: Probability density function
EDTPL: Exact distribution of two independent Lindley random variables (EDTPL)
LD: Lindley distribution
NGLD: New generalized Lindley distribution
NQLD: New quasi Lindley distribution
ATPLD: A three parameter Lindley distribution
− LL: Negative Log Likelihood values
K-S: Kolmogorov–Smirnov
AIC: Akaike information criterion
CAIC: Corrected Akaike information criterion
BIC: Bayesian information criterion
HQIC: Hannan-Quinn information criterion

## References

[1] Abramowitz M, Stegun IA (1970). Handbook of Mathematical Functions, with Formulas, Graphs, and Mathematical Tables.

[2] Ahsanullah M, Shakil M, Kibria BMG (2013). On a probability distribution with fractional moments arising from generalized Pearson system of differential equation and its characterization. Int. J. Adv. Stat Probab.

[3] Ahsanullah M (2017). Characterizations of Univariate Continuous Distributions.

[4] Alkarini SH (2016). A class of Lindley and Weibull distributions. Open J. Stat..

[5] Ali MM, Pal M, Woo J (2007). On the ratio of inverted gamma variates. Aust. J. Statist..

[6] Al-Mutairi DK, Ghitany ME, Kundu D (2013). Inferences on stress-strength reliability from Lindley distributions. Commun. Stat.-Theory Methods.

[7] Cakmakyapan S, Kadilar GO (2016). The lindley family of distributions: properties and applications. Hacettepe J. Math. Stat..

[8] Consortini A, Rigal F (1998). Fractional moments and their usefulness in atmospheric laser scintillation. Pure Appl. Opt.: J. Eur. Opt. Soc. Part A.

[9] Elbatal I, Merovci F, Elgarhy M (2013). A new generalized Lindley distribution. Math. Theory Model..

[10] Emiliano PC, Vivanco MJ, De Menezes FS (2014). Information criteria: How do they behave in different models*?*. Comput. Stat. Data Anal..

[11] Galambos, J., Kotz, S.: Characterizations of Probability Distributions. A Unified Approach with an Emphasis on Exponential and Related Models, Lecture Notes in Mathematics, vol. 675. Springer, Berlin (1978)

[12] Ghitany ME, Atieh B, Nadarajah S (2008). Lindley distribution and its application. Math. Comput. Simul..

[13] Gradshteyn IS, Ryzhik IM (2000). Table of Integrals, Series, and Products.

[14] Innocenti C, Consortini A (2002). Monitoring evolution of laser atmospheric scintillation statistics by use of fractional moments. J. Mod. Opt..

[15] Kagan AM, Linnik YuV, Rao CR (1973). Characterizations Problems in Mathematical Statistics.

[16] Korhonen PJ, Narula SC (1989). The probability distribution of the ratio of the absolute values of two normal variables. J. Stat. Comput. Simul..

[17] Lindley DV (1958). Fiducial distributions and Bayes’ theorem. J. Roy. Stat. Soc. B.

[18] Lee RY, Holland BS, Flueck JA (1979). Distribution of a ratio of correlated gamma random variables. SIAM J. Appl. Math..

[19] Lee, E.T., Wang, J.W.: Statistical methods for survival data analysis. John Wiley & Sons, Inc., Hoboken, New Jersey, 3ed. (2003)

[20] Marsaglia G (1965). Ratios of normal variables and ratios of sums of uniform variables. J. Am. Stat. Assoc..

[21] Mazucheli J, Achcar JA (2011). The Lindley distribution applied to competing risks lifetime data. Comput. Methods Programs Biomed..

[22] Nadarajah S (2005). Products, and ratios for a bivariate gamma distribution. Appl. Math. Comput..

[23] Nadarajah S, Gupta AA (2005). On the product and ratio of Bessel random variables. Int. J. Math. Math. Sci..

[24] Nadarajah S, Gupta AA (2006). On the ratio of logistic random variables. Comput. Stat. Data Anal..

[25] Nadarajah S, Kotz S (2006). On the ratio of Fr´echet random variables. Qual. Quant..

[26] Nagaraja H (2006). Characterizations of Probability Distributions. Springer Handbook of Engineering Statistics.

[27] Pham-Gia T (2000). Distributions of the ratios of independent beta variables and applications. Commun. Stat. Theory Methods.

[28] Press SJ (1969). The t ratio distribution. J. Am. Stat. Assoc..

[29] Pinelis I (2018). Positive-part moments via characteristic functions, and more general expressions. J. Theor. Probab..

[30] Prudnikov AP, Brychkov YA, Marichev OI (1986). Integrals and Series (Volumes 1, 2, and 3).

[31] Shakil M, Kibria BMG (2006). Exact distribution of the ratio of gamma and Rayleigh random variables. Pak. J. Stat. Oper. Res..

[32] Shakil M, Kibria BMG, Chang KC (2008). Distributions of the product and ratio of Maxwell and Rayleigh random variables. Stat. Pap..

[33] Shakil M, Ahsanullah M, Kibria BMG (2018). On the characterizations of Chen’s two-parameter exponential power life-testing distribution. J. Stat. Theory Appl..

[34] Shanker R, Amanuel GH (2013). A new quasi lindley distribution. Int J Stat Syst.

[35] Shanker R, Ghebretsadik AH (2013). A new Quasi Lindley distribution. Int. J. Stat. Syst..

[36] Shanker R, Shukla KK, Shanker R, Leonida TA (2017). A three-parameter Lindley distribution. Am. J. Math. Stat..

[37] Shannon CE (1948). A mathematical theory of communication. Bell Syst. Tech. J..

[38] Tomy L (2018). A retrospective study on Lindley distribution. Biom. Biostat. Int. J.

[39] Weiss GH, Havlin S, Matan O (1989). Properties of noninteger moments in a first passage time problem. J Stat Phys.

